# Multiple Cranial Nerve Palsies Without Limb Weakness: A Rare Cranial Variant of Guillain-Barré Syndrome

**DOI:** 10.7759/cureus.60013

**Published:** 2024-05-09

**Authors:** Laxman Wagle, Alexander Reyes, Rashmita Regmi, Dhiraj R Regmi, Anish Thapa

**Affiliations:** 1 Internal Medicine, Ascension Saint Agnes Hospital, Baltimore, USA; 2 Nursing, Karnali Academy of Health Science, Jumla, NPL; 3 Internal Medicine, Nepalese Army Institute of Health Sciences, Kathmandu, NPL; 4 Internal Medicine, Universal College of Medical Sciences, Bhairahawa, NPL

**Keywords:** albuminocytologic dissociation, bilateral facial nerve palsy, guillain barre’s syndrome (gbs), cranial variant, bilateral limb weakness, cranial nerve palsies

## Abstract

We herein report a case of an unusual variant of Guillain-Barré syndrome (GBS) where the patient presented with multiple bilateral cranial nerve palsies involving nerves V, VII, IX, and X, leading to difficulties with eye closure, eyebrow-raising, chewing, swallowing, and speech. Sensation and motor examination were normal. Bilateral knee reflexes were absent. Lumbar puncture showed cerebrospinal fluid albuminoid-cytologic dissociation. Prompt initiation of plasmapheresis therapy facilitated a successful recovery. This case report underscores the significance of early identification and tailored intervention for atypical GBS presentations, highlighting the potential for improved patient outcomes through targeted management strategies.

## Introduction

Guillain-Barré syndrome (GBS) is a rare acute autoimmune polyneuropathy caused by demyelination of the peripheral nerves. The typical clinical picture in GBS is of ascending paralysis, which starts with weakness and paresthesia in the legs and progressively causes paralysis of both upper and lower limbs, causing tetraparesis [1]. This is usually associated with cranial nerve involvement [1,2]. However, multiple cranial neuropathies without limb weakness can occur, which is a rare variant of GBS, accounting for about 5% of total cases [3]. Wang et al. conducted a retrospective study of 12 cases of multiple cranial neuropathy variant of GBS of which eight patients had an impairment of cranial nerves (CNs) IX and X, three patients had an impairment of CN VII; and the remaining one had impairment of CNs VII, IX, and X [4]. Similarly, Wakerley and Yuki who studied 15 historical cases of this variant noted the involvement of various cranial nerves: CN III (14/15), CN IV (10/15), CN V (5/15), CN VI (13/15), CN VII (12/15), CN IX (14/15), CN X (14/15), CN XI (4/15), and CN XII (5/15) [5].

## Case presentation

**Figure 1:**
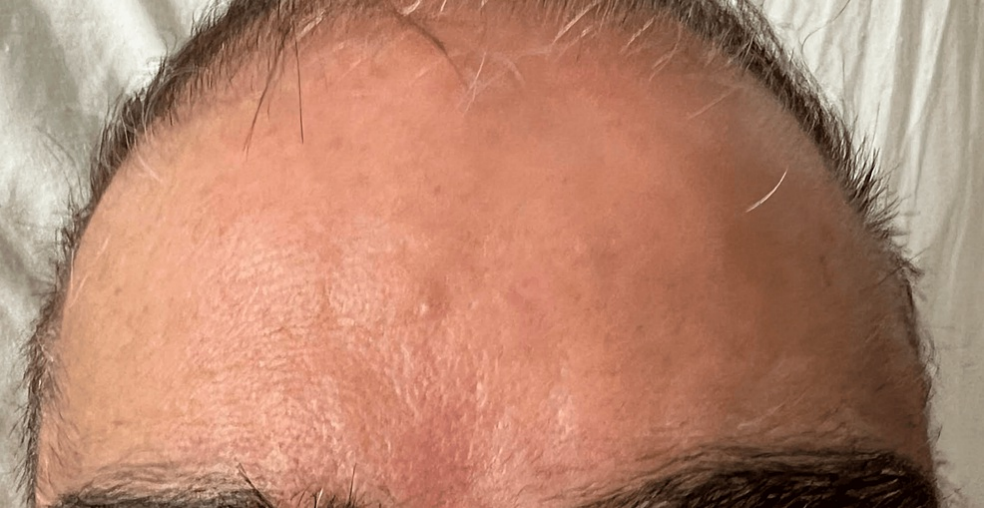
Inability to frown in bilateral facial nerve palsy

We present a case of a 65-year-old male who presented to the emergency department with progressive slurring of speech, left-sided facial numbness, and difficulty closing both eyes for two days. He had recurrent upper respiratory symptoms and was empirically treated for sinusitis with amoxicillin-clavulanate for ten days two weeks prior. He denied fever, neck pain, photophobia, recent sick contact, or travel history. On admission, his vitals were normal except for an elevated blood pressure of 160/73 mmHg. Physical examination revealed multiple bilateral cranial nerve palsies involving nerves V, VII, IX, and X. He displayed slurred speech, difficulty closing both eyes tightly, raising eyebrows (see Figure 1), chewing, and swallowing. Sensation was intact, motor power was 5/5 in all extremities, knee reflexes were absent, and there were no signs of neck rigidity or Kernig's or Brudzinski’s sign. 

He was investigated for viral encephalitis due to his recent viral symptoms and cranial nerve involvement. He was started on empiric acyclovir. Blood tests were unremarkable, including complete blood count, comprehensive metabolic profile, and thyroid function test. MRI brain and CT angiography of the chest findings were unremarkable. Tests for COVID-19, Influenza, HIV, syphilis, Lyme disease, Epstein-Barr virus, and cytomegalovirus were negative. Autoimmune tests were within normal ranges, including antinuclear antibody, myasthenia gravis antibody panel, and ganglioside antibody panel, including GQ1b antibody. The patient developed acute kidney injury after initiation of acyclovir. 

A lumbar puncture revealed cerebrospinal fluid (CSF) albuminoid-cytologic dissociation with a WBC count of 3 and a total protein of 72. Viral encephalitis panels were negative, and CSF culture showed no growth. Despite the absence of limb weakness, the combination of symptoms with cranial nerve involvement and CSF findings raised suspicion for GBS with an atypical presentation. The patient was promptly started on plasmapheresis therapy. Intravenous immunoglobulin (IVIG) was not given due to acute kidney injury. 

**Figure 2:**
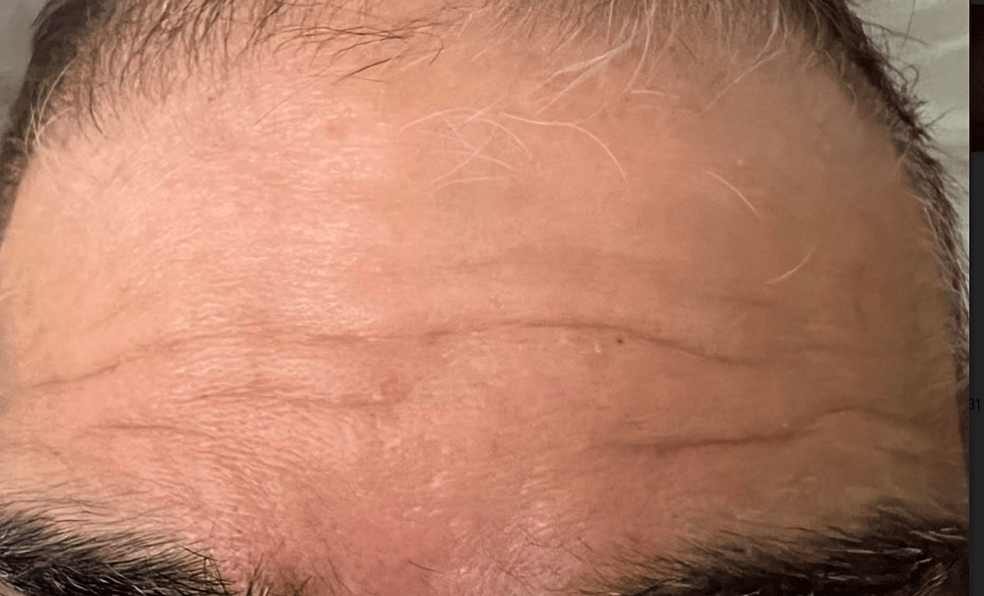
The sequential improvement in frowning following plasmapheresis

**Figure 3:**
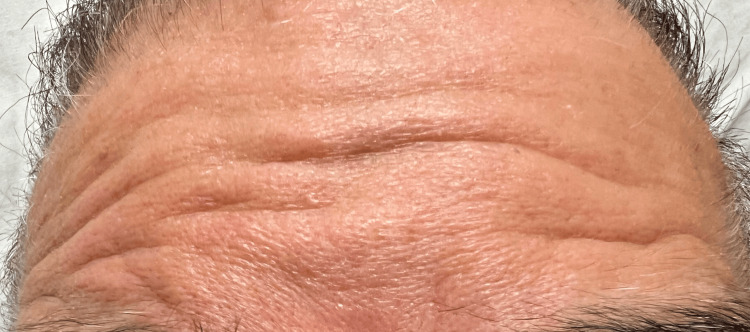
The sequential improvement in frowning following plasmapheresis

Following plasmapheresis initiation, the patient demonstrated gradual improvement in symptoms. The slurring of speech and his ability to frown improved (Figure 2 and Figure 3), and there was an improvement in his ability to close his eyes. Subsequent neurological examination demonstrated improvement in cranial nerve function supporting the diagnosis of GBS. His kidney function also improved after acyclovir was stopped.

## Discussion

GBS is an acute inflammatory demyelinating polyneuropathy that can present with a variety of clinical features; the majority of the patients have ascending paralysis. In a study of 61 patients with GBS, 38 or 62.3% of the 61 patients had cranial nerve palsies and limb involvement. Out of the 38, 25 had multiple cranial nerve palsies, and 13 had a single type of nerve palsy [2]. Different studies showed that 50% to 75% of cases with GBS had cranial nerve involvement. Lφeffel et al. quoted 50%, and Dhadke et al. had 62.5% involvement [1,6]. Most cranial neuropathies in GBS variants present with limb involvement, but multiple cranial neuropathies without limb weakness are rare GBS variants [7]. 

Wakerley et al. proposed that a small subset of patients of GBS spectrum disorders who develop multiple cranial neuropathies without ataxia or limb weakness can not be classified into any known subtype of GBS and should be a distinct subtype, namely polyneuritis cranialis [8]. They concluded that the absence of ataxia was cardinal in separating this disorder from Miller Fisher syndrome (MFS), and the lack of cervical-brachial weakness ruled out the pharyngeal-cervical-brachial weakness subtype of GBS. Facial weakness was reported to be a distinct feature of polyneuritis cranialis [8]. 

In a retrospective review of 12 hospitalized patients with GBS with multiple cranial nerve impairments without limb weakness, none of these patients had an exact permutation as ours [4]. Our patient had bilateral facial nerve involvement along with the involvement of CNs V, IX, and X.

Wang et al. concluded that bilateral CN IX, X, or VII impairment with areflexia or hyporeflexia, nerve conduction abnormalities, and CSF albuminocytologic dissociation support a diagnosis of GBS cranial variant. They noted that albuminocytologic dissociation appeared only in half the cases within the first week and usually appeared in all cases within two to three weeks [4]. In a review of 15 historical cases of polyneuritis cranialis, anti-GQ1b antibodies were negative in five out of eight tested cases [5]. 

Even though our case had albuminocytologic dissociation on the fifth day, focusing on clinical features seems essential for diagnosing this rare entity. Features such as facial weakness and areflexia in the absence of limb weakness, ataxia, and cervical-brachial weakness are important in aiding the diagnostic process.

IVIG has been used to treat the cranial variant of GBS. The recovery time is variable but within two to three months of disease onset in most cases [4,5,9]. Even though non-sucrose-containing IVIGs might have a lower risk of causing acute kidney injury, the risk of renal impairment exists [10]. Furthermore, a randomized controlled trial has shown plasmapheresis to be equivalent to IVIG in reducing the amount of disability at four weeks in patients with GBS [11]. Given acute kidney injury, plasmapheresis was used as the modality of treatment in our case, which caused prompt recovery. 

## Conclusions

Multiple cranial nerve palsies without limb weakness is a rare variant within the Guillain-Barré syndrome spectrum. Maintaining a high index of suspicion and systematically evaluating for this rare cranial variant is crucial for early diagnosis and appropriate management, thus improving patient outcomes. IVIG is the preferred treatment, but in cases with contraindications to IVG such as ours, plasmapheresis can be the modality of treatment. Further studies are needed to confirm the efficacy of plasmapheresis in multiple cranial nerve variants of GBS.
